# A case of successful slide tracheoplasty for long-segment congenital tracheal stenosis in a neonate with a congenital diaphragmatic hernia and Fallot’s tetralogy

**DOI:** 10.1186/s40792-022-01422-9

**Published:** 2022-04-13

**Authors:** Marie Todo, Hiroomi Okuyama, Ryuta Saka, Yuko Tazuke, Takayoshi Ueno, Yoshiki Sawa

**Affiliations:** 1 Department of Pediatric Surgery, Osaka University Graduate School of Medicine, 2-15 Yamadaoka, Suita, Osaka 565-0871 Japan; 2 Department of Pediatric Surgery, National Hospital Organization Fukuyama Medical Center, 4-14-17 Okinogamicho, Fukuyama, Hiroshima 720-8520 Japan; 3 Department of Cardiovascular Surgery, Osaka University Graduate School of Medicine, 2-15 Yamadaoka, Suita, Osaka 565-0871 Japan

**Keywords:** Congenital tracheal stenosis, Slide tracheoplasty, Neonatal surgery

## Abstract

**Background:**

Congenital tracheal stenosis (CTS) is a rare and life-threatening airway disorder, which is often associated with cardiac malformations. Among them, neonatal symptomatic CTS with cardiac malformations has an extremely poor prognosis. In contrast to cardiac malformation, congenital diaphragmatic hernia (CDH) has rarely been associated with CTS. We report a neonatal case in which slide tracheoplasty and intracardiac repair were performed simultaneously for CTS and Fallot’s tetralogy (TOF).

**Case presentation:**

An infant with left CDH and Fallot's tetralogy (TOF) was born by cesarean section at 38 weeks of gestation. At the time of resuscitation, a 2.5 mm (ID) endotracheal tube could only be inserted just below the vocal cords. After repairing the CDH at 3 days of age, planned extubation was performed at 7 days of age. However, the patient required re-intubation due to life-threatening episodes after 2 days of the extubation. Enhanced CT revealed a long segment CTS from the upper trachea to the right bronchus (length of stenosis: 40 mm, minimum inner diameter: 2 mm). At 24 days of age, veno-arterial extracorporeal membrane oxygenation (ECMO) was introduced due to severe respiratory failure. At 28 days of age, slide tracheoplasty and palliative right ventricular outflow tract reconstruction (RVOTR) was performed with cardiopulmonary bypass (CPB). After tracheoplasty, a 3.5 mm tracheal (ID) tube could be placed in the reconstructed trachea in a patient with CTS. ECMO was completed 7 days after the operation. On the 17th day after the operation, he was extubated successfully. He was discharged 5 months after birth with home oxygenation therapy.

**Conclusions:**

We reported the successful simultaneous correction of slide tracheoplasty and palliative RVOTR for a neonate with CDH. ECMO was used for respiratory management before and after surgery.

**Supplementary Information:**

The online version contains supplementary material available at 10.1186/s40792-022-01422-9.

## Background

Long-segment congenital tracheal stenosis (CTS) is a rare and life-threatening congenital airway disorder. Moreover, neonatal CTS is associated with high mortality (70%) [1]. Although CTS is often associated with cardiac anomalies, the association of congenital diaphragmatic hernia (CDH) is rare [2].

We herein report a case of neonatal CDH in which a simultaneous operation of slide tracheoplasty and RVTOR was successfully performed for CTS and Fallot’s tetralogy (TOF).

## Case presentation

A 2837 g male infant with an antenatal diagnosis of left CDH and TOF was born by cesarean section at 38 weeks of gestation. The Apgar scores were 5 at 1 min and 5 at 5 min. At the time of resuscitation, a 2.5 mm (ID) endotracheal tube could only be inserted just below the vocal cords. Therefore, congenital tracheal stenosis was suspected.

**Fig. 1:**
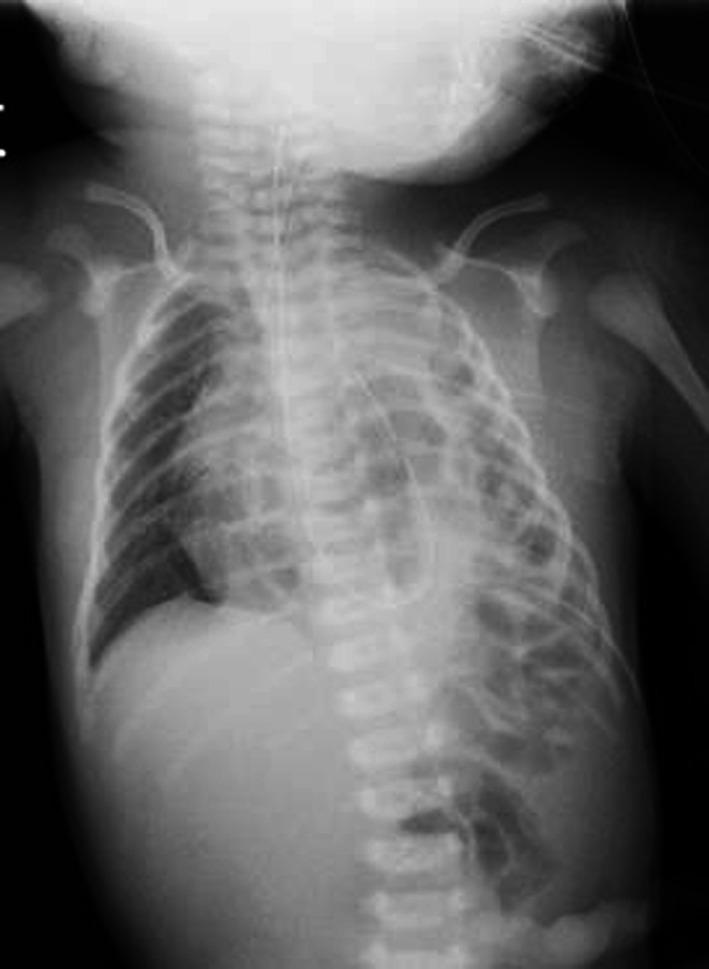
Chest X-ray shows the stomach and intestine in the thoracic cavity. The diagnosis of CDH was confirmed

**Fig. 2:**
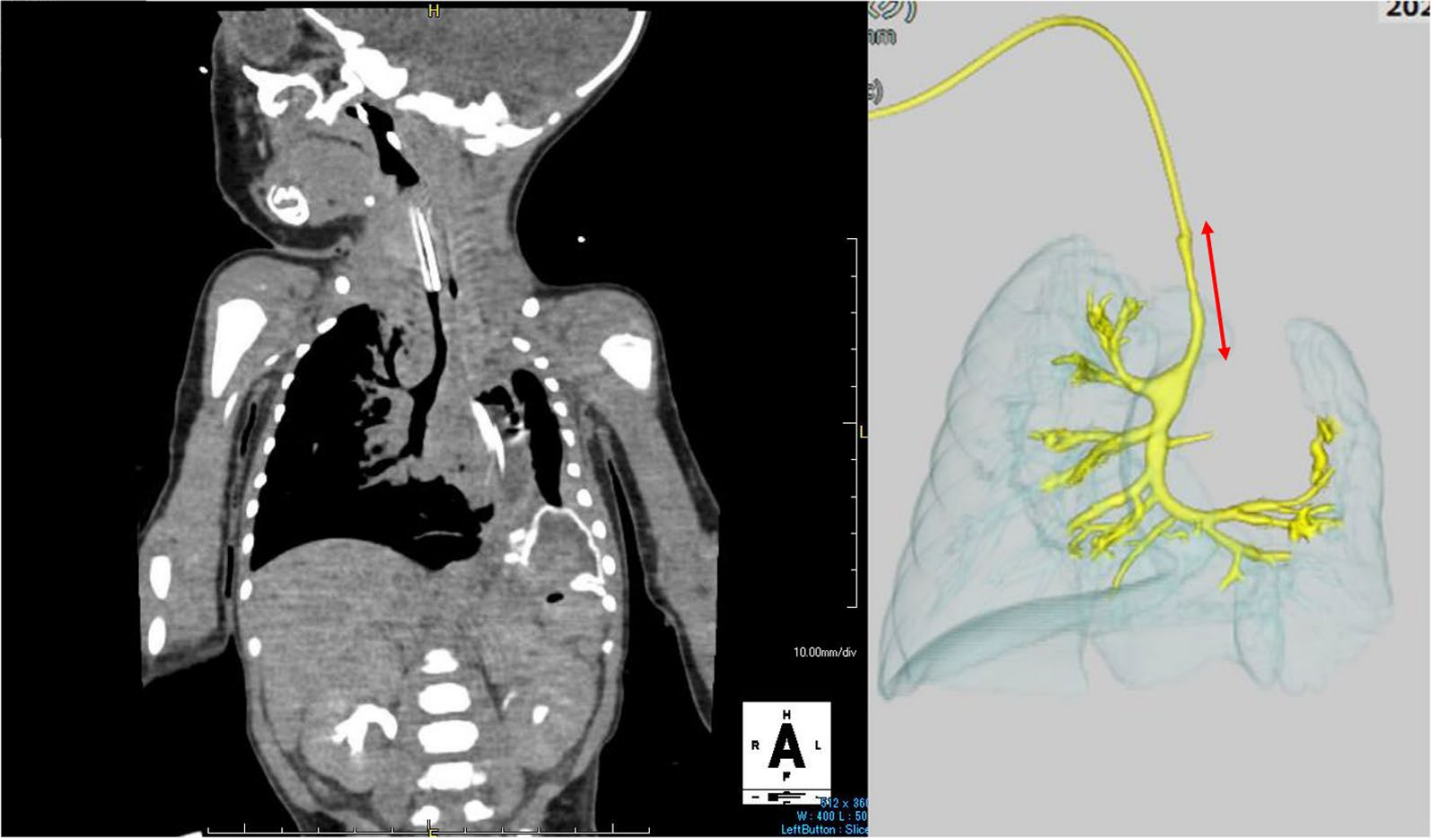
Enhanced CT confirmed the diagnosis of CTS (arrow), and left lung agenesis. The tracheal stenosis was approximately 4 cm in length, extending from upper trachea to the just above right upper lobe bronchus

Chest X-ray confirmed a diagnosis of left CDH (Fig. 1). Echocardiography performed at 2 days of age showed only the right pulmonary artery. After stabilization, he underwent CDH repair at 3 days of age. The diaphragmatic defect was 6 × 4 × 5 cm. After reducing the herniated organs, including the small intestine, the left lobe of the liver, the stomach, and the spleen, the left lung could not be identified in the thoracic cavity. The defect was repaired using a GORE-TEX^®^ Soft Tissue Patch (Thickness: 1 mm). At 7 days after birth, he was extubated successfully. However, he required re-intubation due to progressive respiratory distress at 9 days after birth. In addition to the tracheal edema caused by intubation, increased sputum after long-term intubation was considered to be the cause of obstruction. Enhanced CT revealed long segment CTS with left lung agenesis (Fig. 2). The lung segment in the left thoracic cavity on CT was the lower lobe of the right lung herniated into the left thoracic cavity, and the tracheal branch extending to the left was the right middle lobe branch, not connecting to the lung parenchyma. The tracheal stenosis was approximately 4 cm in length, extending from the upper trachea to the right main bronchus. In addition, it confirmed that the tip of the intubation tube was located just before the tracheal stenosis lesion. Bronchofiberscopy showed complete cartilage rings starting from just below the vocal cord.

At 24 days of age, veno-arterial extracorporeal membrane oxygenation (ECMO) was introduced due to life-threatening airway obstruction. In preoperative management, muscle relaxant was used once when breathing was unstable. At 28 days of age, slide tracheoplasty and palliative right ventricular outflow tract reconstruction (RVOTR) was performed with cardiopulmonary bypass (CPB) (body weight: 2826 g).

**Fig. 3:**
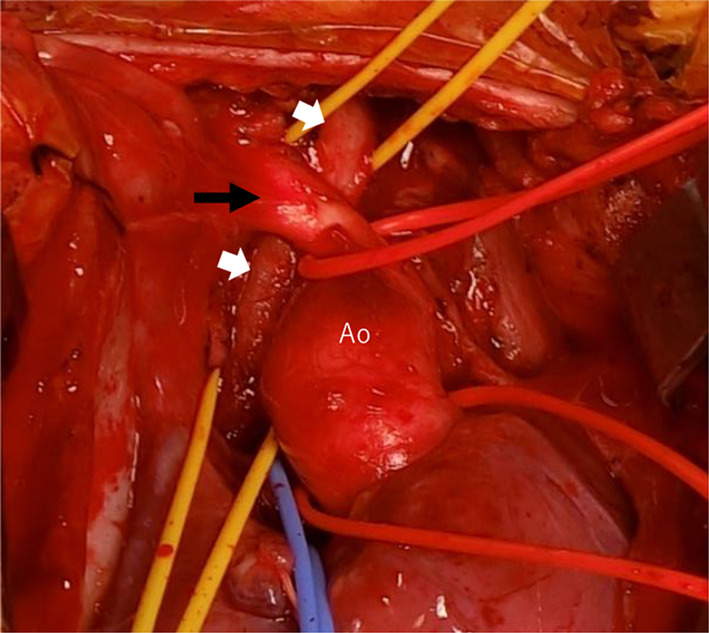
Brachiocephalic artery (black arrow) was located in front of the narrowed trachea (white arrows)

**Fig. 4:**
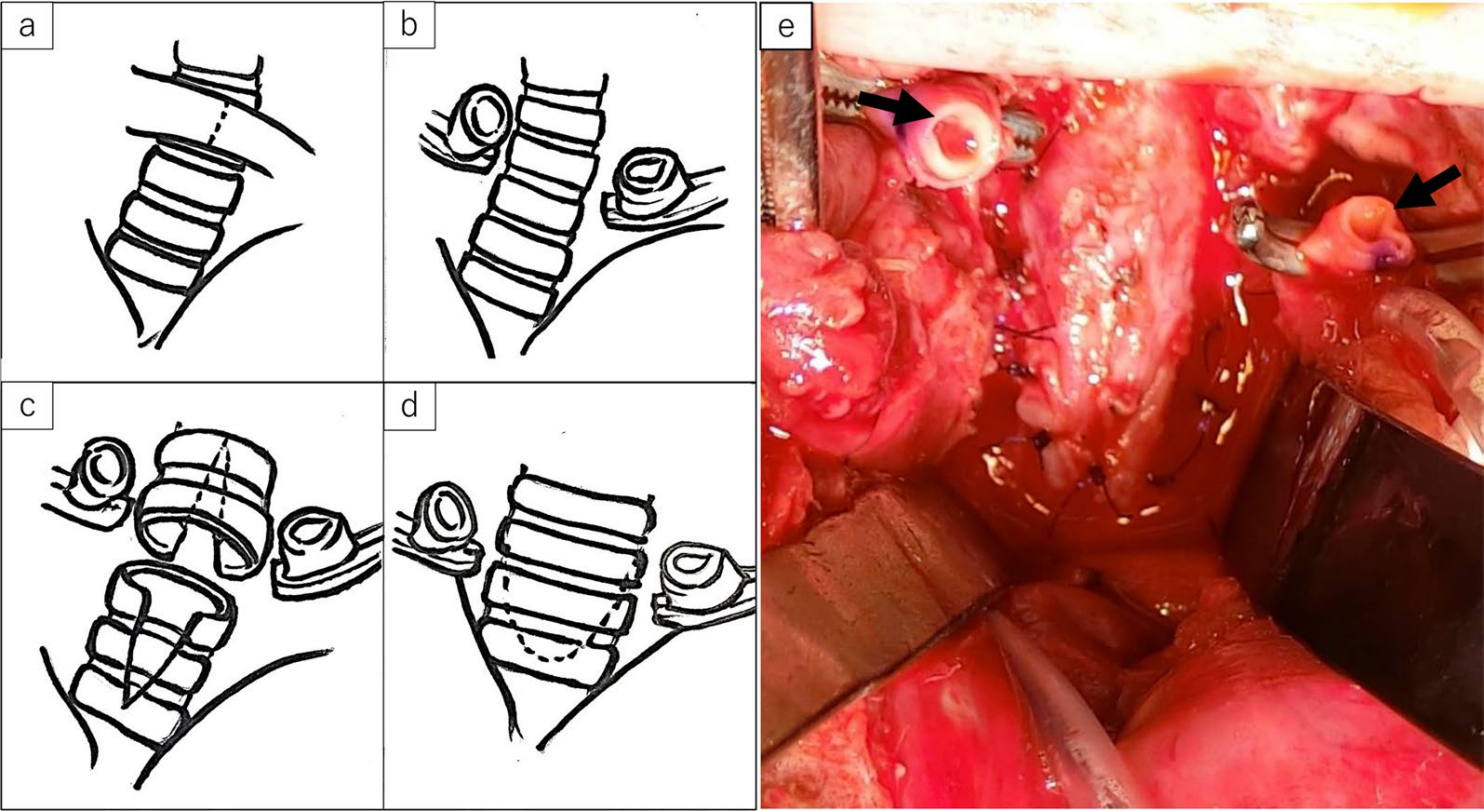
Brachiocephalic artery (arrows) was transected to secure the upper field of slide tracheoplasty, and slide tracheoplasty was performed

The operation was performed through a midline sternotomy plus collar incision. After thoracotomy, ECMO was switched to CPB using the aorta and right atrium cannulation. The ascending aorta, brachiocephalic artery, and trachea were dissected and encircled with vessel loops (Fig. 3). Following the establishment of cardiopulmonary bypass, palliative RVOTR and PDA ligation were performed. The brachiocephalic artery was transected temporarily to secure the upper field of slide tracheoplasty (Fig. 4a, b, Additional file 1). The tracheal stenosis was measured from the upper trachea to just above the carina (length: 40 mm, inner diameter: 2 mm). Transverse division of the trachea was performed in the middle of the stenosis (Fig. 4c). A longitudinal incision of 2 cm in length was made on the posterior side of the proximal trachea and the anterior side of the distal trachea. The two ends were slid to each other, and anastomosis was performed using 5–0 monofilament sutures. After reconstruction, a tracheal tube (ID: 3.5 mm, OD: 4.8 mm) was inserted near the tracheal bifurcation and placed so that the tip exceeded the anastomotic site (Fig. 4d). The brachiocephalic artery was reconstructed, because it was necessary to continue ECMO from the carotid artery after the operation. Then, CPB was successfully discontinued, followed by the reintroduction of ECMO. The operation time was 7 h and 36 min, and the amount of bleeding was 2197 ml (ECC: 1827 ml). As postoperative management, muscle relaxants were used for 14 h postoperatively, and ECMO was completed 7 days after the operation. During ECMO management, 3 days after surgery, a hyperechoic region appeared in the left middle cerebral artery region by head ultrasonography, which was cerebral edema due to cerebral infarction.

**Fig. 5:**
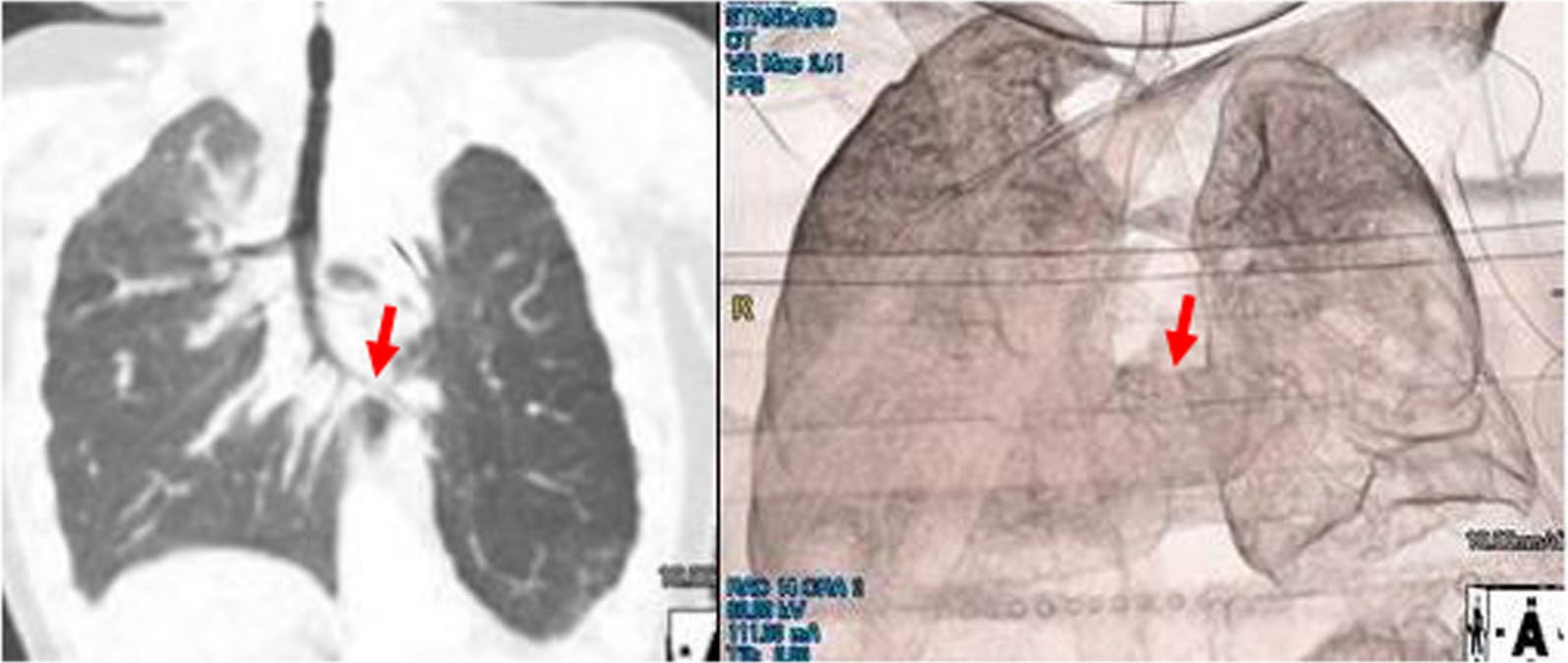
A 4-month-old chest CT image showed no left lung or trachea, and the right lung remained hernia from the dorsal side of the heart into the left thoracic cavity (arrows)

**Fig. 6:**
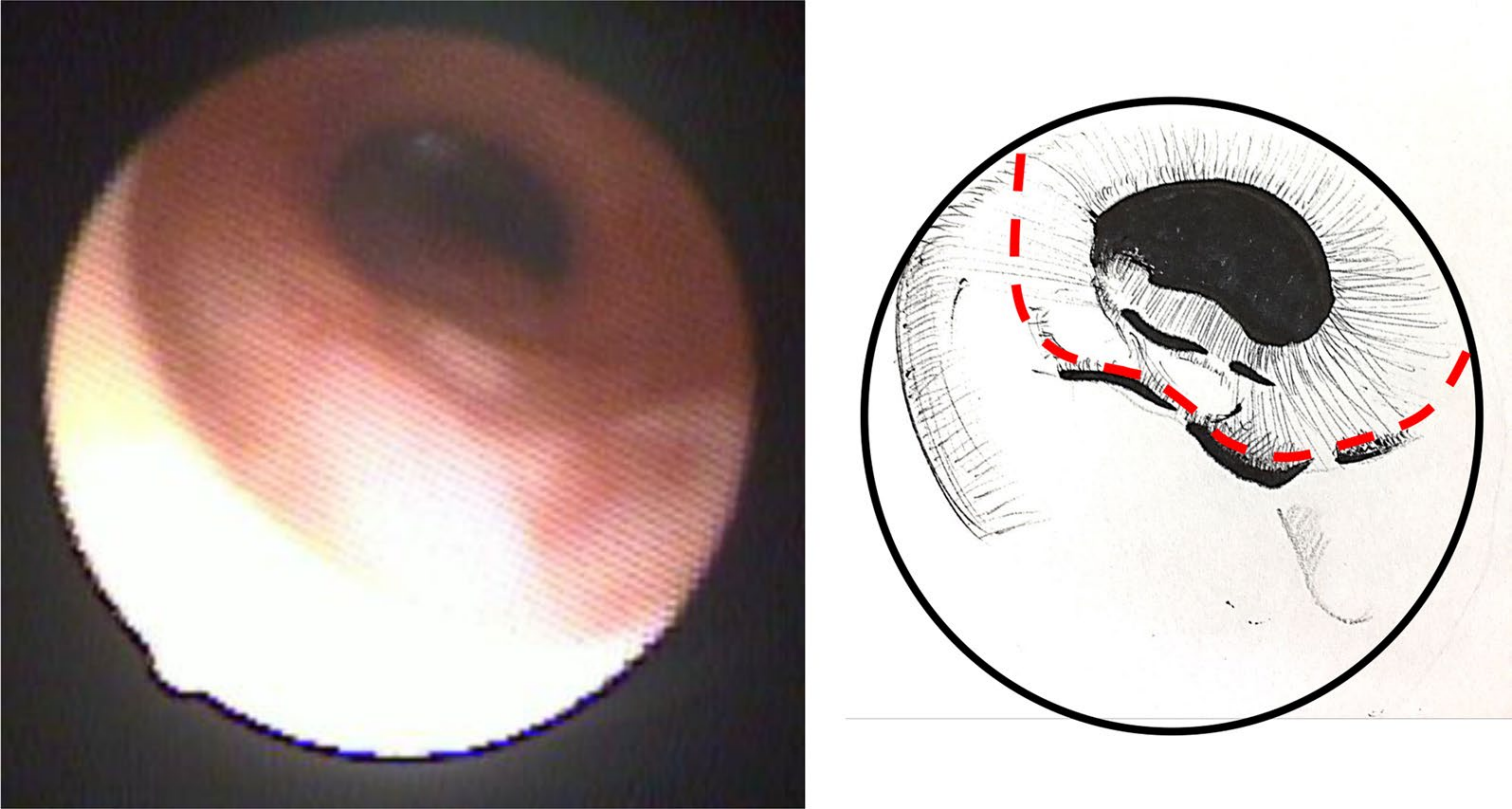
Bronchoscopy at 8 months of age showed no granulation at the site of anastomosis (red dotted line)

On the 17th postoperative day, he was successfully extubated. He was discharged at 5 months of age with home oxygenation therapy and continuous enteral nutrition through a nasogastric tube. Unfortunately, left side cerebral infarction was detected by postoperative head CT, which caused slightly delayed mental development. Six days after extubation, the infant no longer needed oxygen administration, but high-pressure treatment needed to be continued, and oxygen saturation easily decreased during awakening. Three months after the operation, the respiratory condition had settled down, and then preparations for discharge were started. At the time of discharge, it was necessary to continue Nasal High Flow, HOT therapy, and tube feeding at home. A 4-month-old chest CT image 3 months after the operation is shown. A 4-month-old chest CT image showed no left lung or trachea, and the right lung remained hernia from the dorsal side of the heart into the left thoracic cavity (Fig. 5), and bronchoscopy at 8 months of age showed no granulation, tracheomalacia, or re-stenosis after CTS construction (Fig. 6).

## Discussion

Long-segment congenital tracheal stenosis (CTS) is rare, and the incidence has been reported to be 1 in 50,000–60,000 births [3]. The severity of CTS depends on (1) the degree of stenosis, (2) the length of the affected lesion, and (3) the presence of congenital heart disease (CHD) [4–6].

Surgical management is recommended for children with significant respiratory symptoms. However, the morbidity and mortality of CTS requiring surgical intervention in the neonatal period are still high. The mortality rate of CTS associated with CHD is reported to be 53% [1]. In single-lung patients, the mortality rate after tracheoplasty is not increased, ranging from 33 to 65% [1, 7]. Nevertheless, these patients are proven to be more difficult to manage, both preoperatively and postoperatively. The mortality of tracheoplasty in the neonatal period has been reported to be 70% [1].

Recently, slide tracheoplasty, first described by Tsang et al. in 1989, has become a standard surgical procedure for long-segment CTS [8]. This approach is associated with lower morbidity (5%) in comparison to other tracheal reconstruction techniques and can be applied to various anatomical deformities [7, 9].

The advantages of slide tracheoplasty include: (a) reconstructing the trachea with tracheal tissue; and (b) extending the inferior incision to the bronchus in cases with tracheal stenosis [10]. In our CTS patient with left agenesis, slide tracheoplasty was possible, because an incision on the trachea could be extended into the right bronchus.

CTS is frequently associated with cardiovascular anomalies (50–88%), including pulmonary artery sling, tetralogy of Fallot, double aortic arch, and transposition of the great arteries [1]. CTS sometimes complicates unilateral lung agenesis or severe hypoplasia (8–20%) [11–13]. There is only one report of the association of CTS and CDH [14]. In our case, ECMO was extremely effective for respiratory management before and after slide tracheoplasty. To the best of our knowledge, this is the first report of the successful performance of slide tracheoplasty in a neonate with CTS, TOF, and CDH.

## Conclusions

We report a neonatal case in which successful slide tracheoplasty and intracardiac repair were performed simultaneously for CTS and TOF. ECMO was effectively used for respiratory management before and after surgery.

## Supplementary Information

**Additional file 1: **Video. The surgical video of the tracheoplasty part in this case.

## Data Availability

The authors declare that all data in this manuscript are available within the article.

## Abbreviations

CTS: Congenital tracheal stenosis
CDH: Congenital diaphragmatic hernia
TOF: Fallot’s tetralogy
ECMO: Veno-arterial extracorporeal membrane oxygenation
RVOTR: Right ventricular outflow tract reconstruction
CPB: Cardiopulmonary bypass
CHD: Congenital heart disease
